# Enhancing Consistency in Posterior Malleolus Fracture Classification: A Comprehensive Interobserver Reliability Study With 20 Raters Using the Mason & Molloy Classification

**DOI:** 10.7759/cureus.48586

**Published:** 2023-11-09

**Authors:** Theenesh Theyvan Balakrishnan, Ahmad Bilal, Niall Fitzpatrick, Rohan Dahiya, Shahrul Aiman Soelar, Karniza Khalid, Anand Pillai

**Affiliations:** 1 Orthopedics and Traumatology, Wythenshawe Hospital, Manchester, GBR; 2 Trauma and Orthopedics, Wythenshawe Hospital, Manchester, GBR; 3 Trauma and Orthopedics, Manchester NHS Foundation Trust, Manchester, GBR; 4 Research, Clinical Research Center, Hospital Sultanah Bahiyah, Kedah, MYS; 5 Biochemistry, Institute for Medical Research, National Institutes of Health, Kuala Lumpur, MYS; 6 Trauma and Orthopedics, Wythenshawe Hospital, Manchester University NHS Foundation Trust, Manchester, GBR

**Keywords:** interobserver reliability, ankle trauma, classification, posterior malleolus, posterior malleolus fracture

## Abstract

Introduction: Over the past decade, there has been a growing interest in the identification and treatment of posterior malleolus fragments, driven by a better understanding of their significance. The Mason & Molloy (M&M) classification system has emerged as a valuable tool for systematically categorizing these fractures and assisting clinicians in formulating treatment. We aim to assess the interobserver reliability of the M&M classification for posterior malleolus fracture by using 20 raters.

Methodology: The study was conducted at a major foot and ankle referral center in Wythenshawe, Manchester, UK. Thirty-eight Computed Tomography (CT) scans were evaluated by 20 independent raters: 15 general orthopedic and trauma surgeons plus five foot and ankle surgeons. Each rater classified the posterior malleolus fracture according to M&M classification into type 1, 2A, 2B, 3, or not classifiable. Statistical analysis was done with the R software package and SPSS (v26; IBM Corp., Armonk, NY). Fleiss kappa (κ) coefficient with a 95% confidence interval (CI) was applied.

Results: The interobserver agreement was moderate with a global κ value of 0.531 (95% CI: 0.518, 0.544). There were good agreements for identifying type 3 M&M (κ=0.785) and those that are not applicable for M&M classification (κ=0.785). There was a strong correlation between all raters in using M&M classification (T_b_=0.53-0.59) except for Rater 12.

Conclusion: M&M classification remains a valuable tool to guide the management of patients with these subsets of ankle fractures.

## Introduction

Fractures involving the posterior malleolus can manifest independently, although they are frequently concurrent with fractures of the medial and lateral malleoli. Approximately 44%-50% of all ankle fractures involve the posterior malleolus [1-3].

Over the past decade, there has been a growing interest in the identification and treatment of posterior malleolus fragments, driven by a better understanding of their significance. Traditionally, authors have advocated to address these fragments when they encompass more than 25% of the plafond [4]. Treatment suggested then was mostly insertion of screws in an anterior-posterior direction during ankle open reduction and internal fixation (ORIF) [5]. However, in recent times, the increased availability of computed tomography (CT) scans has led to a heightened recognition of the change in posterior malleolus fracture management [6].

Contemporary strategies for stabilizing the posterior malleolus fragment typically involve direct fixation, often utilizing either a posterolateral or posteromedial approach [7-9]. This approach enables reattachment of the posterior inferior tibiofibular ligament (PITFL) to achieve bone-to-bone union and also contributes significantly to the stability of the posterior ankle joint and syndesmosis [10,11].

A study conducted by Jeyaseelan et al. showcased substantial improvement in clinical outcomes over a minimum follow-up period of two years for 320 patients who underwent treatment for posterior malleolus fractures, in contrast to those left untreated. However, the study also highlighted a 10% heightened risk of complications and a twofold increase in re-operation rates, primarily attributed to hardware-related issues [12]. Notable classifications for posterior malleolus fracture were proposed by Haraguchi et al. [13] in 2006, followed by Bartonicek et al. in 2015 [14] and the latest by Mason & Molloy (M&M) in 2017 [15].

The M&M CT-based classification system utilized scans from 121 patients, describing four types based on CT scans, anatomical and patho-mechanical considerations. The four types included are type 1 (extra-articular avulsion of the distal posterior tibial cortex by the PITFL-34% prevalence); type 2A (primary fragment of the posterolateral Volkmann area extending into the incisura-25% prevalence); type 2B (primary fragment of the posterolateral Volkmann area extending into the incisura plus a secondary fragment of the posteromedial aspect of the tibia-21% prevalence); and type 3 (fracture extending across the whole posterior plafond-21% prevalence). The authors proposed a surgical algorithm bound by the classification. They reported an interobserver reliability Cohen kappa (κ) value of 0.919 (between two fellowship-trained foot and ankle surgeons) [15].

The M&M classification system has emerged as a valuable tool for systematically categorizing these fractures and assisting clinicians in formulating informed treatment decisions. However, the consistent application and reliability of this classification system across a bigger number of observers remain subjects of crucial inquiry. Hence, our study aims to assess the interobserver reliability of the M&M classification for posterior malleolus fractures, building upon the foundation laid by previous research.

## Materials and methods

The study was conducted at a major foot and ankle referral center in Wythenshawe, Manchester UK. A retrospective search of patients with ankle fractures treated between January 2022 and January 2023 was performed at the institution. Inclusion criteria were patients 18 years and older who had a preoperative ankle CT scan with a posterior malleolus fracture. Exclusion criteria were previous history of ankle fracture, infection, tumor, or surgery. In addition, posterior pilon fractures [14], were excluded.

CT images of 38 patients were evaluated using the digital imaging software, Picture Archive and Communication Systems (PACS) by 20 independent raters: 15 general orthopedic and trauma surgeons plus five foot and ankle surgeons. The raters were exposed to posterior malleolar classifications described by M&M through a direct briefing session before the evaluation. The evaluation was carried out during a regional physical meeting among orthopedic surgeons at our center. Each rater classified the fracture of the posterior malleolus according to M&M classification into type 1, 2A, 2B, 3, or not classifiable. The first response of each evaluator was compared to obtain the interobserver reliability.

Statistical analysis was done with R software package and SPSS (v26; IBM Corp., Armonk, NY). Fleiss kappa (κ) coefficient with 95% confidence interval (CI) was applied to determine the interobserver agreement. Levels of agreement for κ were estimated as proposed by Landis and Koch [16].

κ values of 0.00 to 0.20 were considered slight agreement; 0.21 to 0.40, fair agreement; 0.41 to 0.60, moderate agreement; 0.61 to 0.80, substantial agreement; and 0.81 to 1.00, almost perfect agreement [16]. Individual interobserver agreement was also assessed to look for correlation between the raters.

## Results

A total of 20 raters evaluated 38 CT pictograms of posterior malleolus fracture using the M&M classification. The interobserver agreement was moderate with a global κ value of 0.531 (95% CI: 0.518, 0.544). There were good agreements for identifying type 3 M&M (κ=0.785) and those that are not applicable for M&M classification (κ=0.785) (Table 1).

**Table 1:** Agreement between 20 raters on 38 slides using Fleiss’ kappa test.

Variables		Fleiss' Kappa value	(95% CI)	P-value
Overall		0.531	(0.518,0.544)	<0.001
Rating category				
	Type 1	0.296	(0.273,0.319)	<0.001
	Type 2A	0.539	(0.516,0.562)	<0.001
	Type 2B	0.460	(0.437,0.484)	<0.001
	Type 3	0.722	(0.699,0.745)	<0.001
	Not applicable	0.785	(0.762,0.808)	<0.001

Individual interobserver agreement was also assessed (Table 2). Overall, there was a strong correlation between all raters in using M&M classification (Tb=0.53-0.59) except for Rater 12.

**Table 2:** Inter-rater agreements among 20 raters. Note: Agreement was assessed with Kendall’s tau-b. **Correlation is significant at the 0.01 level (two-tailed) * Correlation is significant at the 0.05 level (two-tailed)

Rater	1	2	3	4	5	6	7	8	9	10	11	12	13	14	15	16	17	18	19	20
1																				
2	0.83**																			
3	0.84**	0.85**																		
4	0.75**	0.67**	0.74**																	
5	0.66**	0.54**	0.66**	0.82**																
6	0.79**	0.72**	0.77**	0.86**	0.78**															
7	0.81**	0.73**	0.69**	0.67**	0.60**	0.75**														
8	0.77*	0.68**	0.67**	0.74**	0.69**	0.85**	0.86**													
9	0.83**	0.85**	0.74**	0.70**	0.60**	0.79**	0.79**	0.81**												
10	0.86**	0.80**	0.81**	0.77**	0.68**	0.85**	0.79**	0.82**	0.83**											
11	0.57**	0.60**	0.55**	0.64**	0.66**	0.71**	0.60**	0.72**	0.69**	0.63**										
12	0.18	0.14	0.11	0.26	0.17	0.27*	0.10	0.13	0.15	0.14	0.13									
13	0.71**	0.80**	0.78**	0.61**	0.51**	0.68**	0.65**	0.61**	0.75**	0.73**	0.53**	0.17								
14	0.83**	0.84**	0.80**	0.79**	0.67**	0.76**	0.77**	0.79**	0.82**	0.76**	0.57**	0.12	0.74**							
15	0.80**	0.69**	0.72**	0.83**	0.75**	0.94**	0.80**	0.87**	0.81**	0.85**	0.71**	0.25	0.67**	0.74**						
16	0.63**	0.74**	0.65**	0.76**	0.59**	0.74**	0.56**	0.59**	0.69**	0.70**	0.58**	0.32*	0.67**	0.71**	0.72**					
17	0.77**	0.69**	0.74**	0.80**	0.72**	0.85**	0.74**	0.79**	0.75**	0.80**	0.57**	0.22	0.79**	0.82**	0.83**	0.66**				
18	0.72**	0.71**	0.66**	0.69**	0.56**	0.69**	0.76**	0.80**	0.79**	0.71**	0.61**	0.08	0.68**	0.77**	0.74**	0.61**	0.70**			
19	0.73**	0.71**	0.65**	0.81**	0.64**	0.73**	0.71**	0.78**	0.77**	0.71**	0.67**	0.14	0.60**	0.83**	0.74**	0.73**	0.74**	0.77**		
20	0.81**	0.82**	0.78**	0.78**	0.66**	0.75**	0.75**	0.78**	0.81**	0.74**	0.56**	0.10	0.73**	0.99**	0.73**	0.72**	0.81**	0.79**	0.82**	

## Discussion

The assessment of interobserver reliability is pivotal in validating the clinical utility of any classification system, as it underpins consistent treatment decisions and patient management. Our findings revealed that the interobserver agreement for M&M classification was moderate with a global κ value of 0.531 (95% CI: 0.518, 0.544). This is comparable with previous studies in the literature. Mustafa et al. compared all the three classifications mentioned earlier and the M&M classification had moderate interobserver reliability with a Fleiss κ value of 0.541 (SE 0.098). This was a study with nine raters [17]. Another paper by Morales et al. also showed moderate interobserver reliability for the M&M classification with a global κ value of 0.54 (95% CI: 0.47-0.62), rated by six evaluators [18]. It is also worth mentioning a study by Kleinertz et al. with four raters which presented Fleiss’ κ value of 0.724 (95% CI: 0.674-0.774) [19].

While the interobserver reliability is moderate (two papers) and substantial (one paper), the number of raters in those studies is a point of paramount significance. A low number has the potential to yield higher rates of agreement among the raters and vice versa. In our study, we had 20 raters, which to our knowledge is the most in the literature for M&M classification. This shows that even with such a large number of raters, the interobserver reliability still remains moderate. It may not seem to be the most ideal classification system to be used, but it still carries a decent role among the larger group of observers.

Previous reliability and reproducibility studies have suggested that the observers’ experience improves the reliability of a classification system [20-22]. The intricate nature of posterior malleolus fractures, characterized by diverse fracture patterns inherently challenges a consistent classification [23,24]. This complexity likely contributes to the variability in interpretations observed across different observers. Additionally, the observers' varying levels of familiarity with the M&M classification system may have played a role in the degree of agreement.

Our study found that there is a correlation between the rater’s experience and interobserver agreement of the Mason & Malloy classification, contrary to previous papers on this subject [17,18]. Rater no. 12, a junior orthopedic surgeon, ranked lowest in agreement with the rest. We postulate that training level, exposure, and frequency of usage of this classification do influence interobserver reliability. In their landmark paper, Mason et al. who conducted the study with an agreement analysis, reported almost perfect interobserver agreement (κ = 0.92) [15]. However, the blinded evaluation was performed by two fellowship-trained senior foot and ankle surgeons.

It is worth highlighting another significant finding in our analysis. Type 1 M&M performed poorly in comparison to other types for the interobserver agreement. Type 3 and the ones that fell under the unclassifiable category had good identification agreement by the 20 raters. This can be attributed to the lack of a clear definition of type 1 M&M in its original description [15]. It is also not uncommon for extra-articular cortical avulsion of the distal posterior tibia to be variable in size and number of fragments. There may also be some crossover to type 2A. Further direction to improve the classification is probably by subdivision of type 1 with emphasis on the fracture fragment size.

Our study is not without its limitations. The sample size was obtained through convenience sampling. Whilst it offers simplicity in data collection, it may over or underrepresent certain types of fractures. It may not reflect the entire posterior malleolus fracture geometry in the wider group of ankle fractures that are present in our institution. We also did not perform a comparison across the other classification systems for posterior malleolus fracture, namely the ones by Haraguchi et al. and Bartonicek et al. This was to ensure that our focus is the one classification that is widely used at our center.

Moving forward, strategies to improve interobserver reliability merit exploration. Standardized educational interventions, coupled with rigorous training in the application of the M&M classification system, have the potential to align observers' interpretations and reduce variability. Additionally, investigating the link between interobserver reliability and patient outcomes remains a promising avenue for understanding the clinical implications of classification consistency.

## Conclusions

Our study contributes valuable insight into the interobserver reliability of the M&M classification for posterior malleolus fractures among a large number of evaluators. The moderate level of agreement observed among the 20 raters here underscores the constantly essential need for refinement in posterior malleolus fracture classification. However, in the interim of producing a more ideal classification system, M&M classification remains a valuable tool to guide the management of patients with these subsets of ankle fracture. In the bigger picture, a concerted effort toward enhancing the reliability of classification systems is important. This ensures that treatment decisions are based on accurate, consistent and easily communicable categorizations, ultimately improving outcomes for patients, especially the ones with posterior malleolus fracture.

## References

[1] Switaj PJ, Weatherford B, Fuchs D, Rosenthal B, Pang E, Kadakia AR (2014). Evaluation of posterior malleolar fractures and the posterior pilon variant in operatively treated ankle fractures. Foot Ankle Int.

[2] Court-Brown CM, McBirnie J, Wilson G (1998). Adult ankle fractures--an increasing problem?. Acta Orthop Scand.

[3] Koval KJ, Lurie J, Zhou W (2005). Ankle fractures in the elderly: what you get depends on where you live and who you see. J Orthop Trauma.

[4] Harper MC, Hardin G (1988). Posterior malleolar fractures of the ankle associated with external rotation-abduction injuries. Results with and without internal fixation. J Bone Joint Surg Am.

[5] Odak S, Ahluwalia R, Unnikrishnan P, Hennessy M, Platt S (2016). Management of posterior malleolar fractures: a systematic review. J Foot Ankle Surg.

[6] Ferries JS, DeCoster TA, Firoozbakhsh KK, Garcia JF, Miller RA (1994). Plain radiographic interpretation in trimalleolar ankle fractures poorly assesses posterior fragment size. J Orthop Trauma.

[7] Choi JY, Kim JH, Ko HT, Suh JS (2015). Single oblique posterolateral approach for open reduction and internal fixation of posterior malleolar fractures with an associated lateral malleolar fracture. J Foot Ankle Surg.

[8] Tornetta P, 3rd 3rd, Ricci W, Nork S, Collinge C, Steen B (2011). The posterolateral approach to the tibia for displaced posterior malleolar injuries. J Orthop Trauma.

[9] Assal M, Ray A, Fasel JH, Stern R (2014). A modified posteromedial approach combined with extensile anterior for the treatment of complex tibial pilon fractures (AO/OTA 43-C). J Orthop Trauma.

[10] Ebraheim NA, Taser F, Shafiq Q, Yeasting RA (2006). Anatomical evaluation and clinical importance of the tibiofibular syndesmosis ligaments. Surg Radiol Anat.

[11] Gardner MJ, Brodsky A, Briggs SM, Nielson JH, Lorich DG (2006). Fixation of posterior malleolar fractures provides greater syndesmotic stability. Clin Orthop Relat Res.

[12] Jeyaseelan L, Bua N, Parker L (2021). Outcomes of posterior malleolar fixation in ankle fractures in a major trauma centre. Injury.

[13] Haraguchi N, Haruyama H, Toga H, Kato F (2006). Pathoanatomy of posterior malleolar fractures of the ankle. J Bone Joint Surg Am.

[14] Bartoníček J, Rammelt S, Tuček M (2017). Posterior malleolar fractures: changing concepts and recent developments. Foot Ankle Clin.

[15] Mason LW, Marlow WJ, Widnall J, Molloy AP (2017). Pathoanatomy and associated injuries of posterior malleolus fracture of the ankle. Foot Ankle Int.

[16] Landis JR, Koch GG (1977). The measurement of observer agreement for categorical data. Biometrics.

[17] Rashid MS, Islam R, Marsden S, Trompeter A, Teoh KH (2023). Validation of three classification systems for posterior malleolus fractures of the ankle. Eur J Orthop Surg Traumatol.

[18] Morales S, Massri-Pugin J, Mery P, Palma J, Filippi J, Villa A (2023). Posterior malleolar fracture assessment: an independent interobserver and intraobserver validation of three computed tomography-based classifications. J Am Acad Orthop Surg Glob Res Rev.

[19] Kleinertz H, Mueller E, Tessarzyk M, Frosch KH, Schlickewei C (2022). Computed tomography-based classifications of posterior malleolar fractures and their inter- and intraobserver reliability: a comparison of the Haraguchi, Bartoníček/Rammelt, and Mason classifications. Arch Orthop Trauma Surg.

[20] Schipper IB, Steyerberg EW, Castelein RM, van Vugt AB (2001). Reliability of the AO/ASIF classification for pertrochanteric femoral fractures. Acta Orthop Scand.

[21] Urrutia J, Zamora T, Yurac R (2017). An independent inter- and intraobserver agreement evaluation of the AOSpine subaxial cervical spine injury classification system. Spine (Phila Pa 1976).

[22] Urrutia J, Zamora T, Yurac R (2015). An independent interobserver reliability and intraobserver reproducibility evaluation of the new AOSpine Thoracolumbar Spine Injury Classification System. Spine (Phila Pa 1976.

[23] Macko VW, Matthews LS, Zwirkoski P, Goldstein SA (1991). The joint-contact area of the ankle. The contribution of the posterior malleolus. J Bone Joint Surg Am.

[24] Terstegen J, Weel H, Frosch KH, Rolvien T, Schlickewei C, Mueller E (2023). Classifications of posterior malleolar fractures: a systematic literature review. Arch Orthop Trauma Surg.

